# Single‐isocenter hybrid IMRT plans versus two‐isocenter conventional plans and impact of intrafraction motion for the treatment of breast cancer with supraclavicular lymph nodes involvement

**DOI:** 10.1120/jacmp.v16i4.5188

**Published:** 2015-07-08

**Authors:** Ahmad Amoush, Eric Murray, Jennifer S. Yu, Ping Xia

**Affiliations:** ^1^ Department of Radiation Oncology Georgia Regents University Augusta GA; ^2^ Department of Radiation Oncology Cleveland Clinic Cleveland OH USA

**Keywords:** breast treatment, hybrid IMRT, single‐isocenter, two‐isocenter

## Abstract

The purpose of this study was to compare the single‐isocenter, four‐field hybrid IMRT with the two‐isocenter techniques to treat the whole breast and supraclavicular fields and to investigate the intrafraction motions in both techniques in the superior direction. Fifteen breast cancer patients who underwent lumpectomy and adjuvant radiation to the whole breast and supraclavicular (SCV) fossa at our institution were selected for this study. Two planning techniques were compared for the treatment of the breast and SCV lymph nodes. The patients were divided into three subgroups according to the whole breast volume. For the two‐isocenter technique, conventional wedged or field‐within‐a‐field tangents (FIF) were used to match with the same anterior field for the SCV region. For the single‐isocenter technique, four‐field hybrid IMRT was used for the tangent fields matched with a half blocked anterior field for the SCV region. To simulate the intrafraction uncertainties in the longitudinal direction for both techniques, the treatment isocenters were shifted by 1 mm and 2 mm in the superior direction. The average breast clinical tumor volume (CTV) receiving 100% ($V_{100\%}$) of the prescription dose (50 Gy) was 99.3%±0.5% and 96.4%±1.2% for the for two‐isocenter and single‐isocenter plans (p<0.05), respectively. The breast CTV receiving 95% of the prescription dose ($V_{95\%}$) was close to 100% in both techniques. The average breast CTV receiving 105% ($V_{105\%}$) of the prescription dose was 32.4%±19.3% and 23.8%±13.3% (p=0.08). The percentage volume of the breast CTV receiving 110% of the dose was 0.4%±1.2% in the two‐isocentric technique vs. 0.1%±0.2% in the single‐isocentric technique. The average uniformity index was 0.91±0.02 vs. 0.91±0.01 in both techniques (p=0.04), but had no clinical impact. The percentage volume of the contralateral breast receiving a dose of 1 Gy was less than 2.3% in small breast patients and insignificant for medium and large breast sizes. The percentage of the total lung volume receiving g>20 Gy ($V_{20\text{Gy}}$) and the heart receiving >30 Gy ($V_{30\text{Gy}}$) were 13.6% vs. 14.3% (p=0.03) and 1.25% vs. 1.2% (p=0.62), respectively. Shifting the treatment isocenter by 1 mm and 2 mm superiorly showed that the average maximum dose to 1 cc of the breast volume was 55.5±1.8 Gy and 58.6±4.3 Gy in the two‐isocentric technique vs. 56.4±2.1 Gy and 59.1±5.1 Gy in the single‐isocentric technique (p=0.46, 0.87), respectively. The single‐isocenter technique using four‐field hybrid IMRT approach resulted in comparable plan quality as the two‐isocentric technique. The single‐isocenter technique is more sensitive to intrafraction motion in the superior direction compared to the two‐isocentric technique. The advantages of the single‐isocenter include elimination of isocentric errors due to couch and collimator rotations and reduction in treatment time. This study supports consideration of a single‐isocenter four‐field hybrid IMRT technique for patients undergoing breast and supraclavicular nodal irradiation.

PACS number: 87.55.D, 87.55.de, 87.55.dk,

## I. INTRODUCTION

Breast conserving surgery (BCS) followed by adjuvant radiotherapy provides excellent cosmetic outcomes, with survival rates equal to those from total mastectomy.^1^, ^2^, ^3^ Irradiation of the supraclavicular fossa is an important component of breast irradiation for many patients.

Three‐field technique with two‐isocenter is a commonly used technique to treat the whole‐breast and the supraclavicular axillary (SCV) region. In this technique, opposing tangent fields are matched with a supraclavicular field using a half beam of the SCV field and couch and collimator rotations on the tangent beams.^4^, ^5^, ^6^, ^7^ For linear accelerators with multileaf collimators (MLC), Lu et al.^(8)^ described in details the three‐fields matching technique. They used the couch and collimator rotations to match with the anterior half‐beam blocked SCV field. The posterior borders of the tangent fields were defined by MLC to mimic the corner block. Couch and collimator rotations and patient repositioning during treatment increase the patient setup time and the potential intrafractional patient movement.

Another solution for the three‐field technique is the single‐isocenter approach, which requires the use of half‐beam blocks for both the tangential and SCV fields. A linear accelerator with asymmetric jaws and a large MLC field size can be used for this technique for most patients. The advantages of the single‐isocenter are eliminating the couch movement and shortening the treatment time. However, only half of the field length can be used (typically maximum of 20 cm field size), which may not be adequate for some patients. This technique may also result in a steep dose gradient at the match‐line between the tangent fields and the supraclavicular field. For patients requiring a field size greater than 20 cm length, the use of the two‐isocenter technique can be chosen. Because the two‐isocentric technique may not be performed frequently in institutions that typically use mono‐isocentric technique, unfamiliarity with this technique may lead to errors in radiation delivery. Alternatively, the single‐isocenter approach can be shifted inferiorly below the match line, and the supraclavicular field and the tangents then can be matched only on the patient's skin without perfect three‐dimensional geometric match.^(9)^

Breast radiation is traditionally planned with a 3D conformal technique (3D CRT) with wedges to compensate for the differential thickness across the breast. 3D CRT improves the local control, but normal tissue toxicity remains a concern.^10^, ^11^, ^12^ Intensity‐modulated radiotherapy (IMRT) is an alternative technique to improve dose homogeneity and decrease normal tissue irradiation. IMRT can achieve better dose homogeneity within the breast and reduce the maximum dose compared to 3D CRT with standard wedges.^(11)^ IMRT techniques can be implemented with either a simple “field‐in‐field” forward planning or complex inverse planning approach. Mihai et al.^(13)^ investigated the dosimetric differences between inverse and forward breast IMRT planning and reported no significant clinical differences, so both techniques can be considered for breast IMRT.

Mayo et al.^(14)^ studied five planning techniques for 10 breast patients: conventional wedged‐field tangents (Tangents), forward‐planned field‐within‐a‐field tangents (FIF), IMRT‐only tangents (IMRT tangents), conventional open plus IMRT tangents (four‐field hybrid), and conventional open plus IMRT tangents with two anterior oblique IMRT beams (six‐field hybrid). They concluded that the four‐field hybrid technique is a viable class solution.

To investigate the organ motion effect on IMRT treatment, Jain et al.^(15)^ used daily CBCT imaging to quantify three‐dimensional (3D) organ/patient motions during whole‐breast IMRT. They concluded that the IMRT dose homogeneity was superior at planning, and remained superior throughout the treatment course despite motion sensitivity, confirming the benefits of IMRT for the patients studied. Michalski et al.^(16)^ provided a comprehensive summary of the literature relating to the magnitude of motion during radiation therapy for a breast cancer patient. The magnitudes of the intrafraction motion are 1.19, 1.26, and 1.82 mm in central lung distance (CLD), central beam edge to skin distance (CBESD), and in craniocaudal distance (CCD), respectively. For interfraction motions, the average movements are 2.21, 1.9, 2.2, 2.6, and 3.18 in CLD, central irradiated width (CIW), CBESD, CCD, and central breast distance (CBD), respectively.^(16)^

In this study, we proposed to use a single‐isocenter technique with four‐field hybrid IMRT as an equivalent method to the conventional two‐isocenter technique to treat the whole breast with the supraclavicular lymph nodes. We also investigated the effect of the intrafraction motion in both techniques by simulating the motions in the treatment planning system in the superior direction.

## II. MATERIALS AND METHODS

### A. Patient selection

A total of 15 patients, who had undergone breast conserving surgery (BCS), were retrospectively selected for this study according to the breast separation. The patients received three‐field treatment using wedged‐field and field‐within‐a‐field tangents (FIF) for the opposing tangential and a slightly oblique anterior field for the SCV region. Eight patients had right‐sided breast cancer and seven had left‐sided breast cancer. The separation between the medial and lateral aspect of the breast were grouped to small, medium, and large (Table 1). All the patients were treated with 6 and 10 MV photons on Siemens ARTISTE linear accelerator (Siemens Medical Solutions USA, Inc., Malvern, PA). Weekly megavoltage (MV) imaging was acquired to verify patient positioning and AlignRT (Vision RT, London, UK) was used for daily patient setup. The prescription dose was 50 Gy in 25 fractions to the breast and 46 Gy to 50 Gy to the supraclavicular lymph nodes.

**Table 1:** Breast dimensions for the three groups of patients. The separation is defined between the medial and lateral aspect of the breast

	*Small Separation (16–19 cm)*	*Medium Separation (19–22 cm)*	*Large Separation (22–26 cm)*
Average Separation SD/(cm)	18.82 (0.19)	21.27 (0.23)	24.09 (0.13)

SD=standard deviation.

### B. Field setup

The patients were simulated on Brilliance CT Big Bore (Philips Medical Systems, Eindhoven, The Netherlands) with 3 mm slice thickness. The patient's arms were elevated in a forearm immobilization device. The treatment field angles and sizes were designed at the time of simulation with the assist of CT images. Specifically, the tangent fields were extended inferiorly 2 cm below the inframammary fold, superiorly to the head of the clavicle, laterally to the mid axillary line, and medially to the midline of the chest. The supraclavicular field was matched to the tangential fields and the shoulder joint was shielded by MLCs.

**Figure 1:**
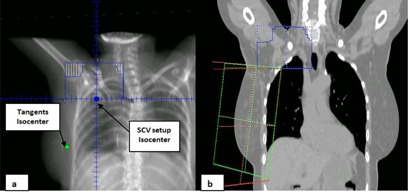
Three‐field setup using two‐isocenter technique. (a) The digitally reconstructed radiographs (DRR) of the coronal beam's eye view (BEV) showing the positions of the tangents and SCV isocenters. The MLC was used to shield the trachea and shoulder joint. (b) The coronal view of the planning CT showing the matching between the SCV field (blue) and tangent field borders (red and green).

All patients were treated in a supine position with two‐isocenter technique using an ARTISTE machine equipped with asymmetric jaws and MLCs. The angles of the fields were selected according to the international electrotechnical commission (IEC) standards. The SCV region was treated with a half‐beam blocked field (Y jaw) with a gantry angle of 10°–15° away from the contralateral breast to avoid the trachea, esophagus, and spinal cord, at 100 cm of the source‐to‐surface distance (SSD). The breast was treated with two tangents. The collimator angle and the table angle of the tangents at a given gantry angle were selected to match the superior border of the tangents with the inferior border of the supraclavicular field. The number of degrees by which the couch and collimator rotated was dependent on the breast dimensions, but is usually within 8°. The tangential fields were angled so that the beam central axes were not opposed but separated by slightly more than 180° to eliminate beam divergence into the lung. Figure 1(a) shows a digitally reconstructed radiograph (DDR) of the beam's eye view (BEV) for the positions of the isocenters, and Fig. 1(b) shows beam arrangements in the coronal view. The posterior borders of the tangents were reduced, when necessary, by the posterior jaws to reduce the volume of lung included in the treatment. For treatments, the SCV field was treated first. Then, isocenter was manually moved to treat the tangents.

**Figure 2:**
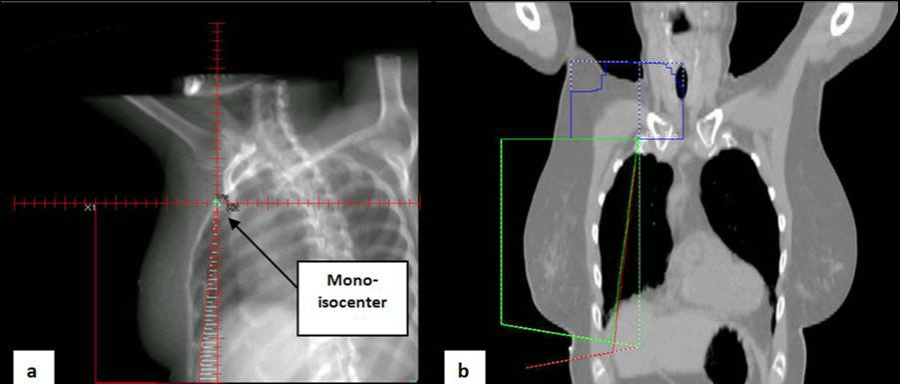
Three‐field setup using single‐isocenter technique. (a) DRR of the coronal BEV showing the position of the isocenter at a depth of 3 cm. Half‐beam blocked fields were used for the tangents and SCV. The MLC was used to mimic the two‐isocentric technique fields to reduce the volume of lung included in the treatment. (b) The coronal view of the planning CT showing the match between the SCV field (blue) and tangent field borders (red and green). For the supraclavicular field, the area outside the solid blue line represents areas shielded by the MLCs.

The same patients were replanned using a single‐isocenter technique. The beam arrangements were the same as in the two‐isocenter technique. Two additional IMRT beams were added to the tangential fields. The collimator and couch rotations were set to zero. The tangent and SCV fields were matched using half‐beam blocks and the isocenter was placed on the edge of each respective field at depth of 3 cm (Fig. 2(a)). To avoid beam divergence into the lung and to mimic the tangents of the two‐isocenter technique, the open tangential beams were shaped by MLCs (Fig. 2(b)).

### C. Treatment planning

The patients were treated with the two‐isocenter technique using wedged‐field and FIF technique for the tangent breast fields and an oblique anterior field for the SCV. The lungs and heart were contoured. Treatment plans were designed on Pinnacle 9.0 treatment planning system (Philips Radiation Oncology Systems, Fitchburg, WI). Treatment goals were: 1) to achieve a uniform dose distribution to the entire breast; 2) to keep the maximum dose <110% of the prescribed dose; 3) to limit the volume of the heart receiving 30 Gy<5%; 4) to limit the dose to the ipsilateral lung; and 5) to limit the dose to the contralateral breast (<1Gy).

For the single‐isocenter technique, the fields were rearranged as described under the field setup section. To generate a breast clinical tumor volume (CTV), the prescription isodose line from the two‐isocenter plan was converted into a contour. Then, the CTV was retracted by 5 mm from the skin surface, as recommended by Saibishkumae et al.^(17)^ The hybrid IMRT plan for the single‐isocenter technique was optimized with direct machine parameter optimization (DMPO) for the IMRT fields and beam weight optimization for the open tangents. For the IMRT beams, the number of segments ranged between 10 to 12 segments with a minimum segment size of $6\;{\text{cm}}^{2}$. The clinically approved two‐isocentric plans were used as a benchmark to evaluate the hybrid IMRT plans.

### D. Intrafraction motion analyses

Intrafraction motion in the superior direction was compared between both planning techniques to investigate hot spots and sensitivity to motion. Intrafraction motions in the lateral and vertical directions were not included in this study as they have minimum dosimetric impact due to the large margins (flash) in both directions. Shifts of 1 mm and 2 mm were selected in this study, based on the review paper by Michalski et al.^(16)^ The shifts were simulated in Pinnacle 9.0 by moving the treatment isocenter in the superior direction and recalculating the dose. The motion in the superior direction in the single‐isocenter technique is primarily due to the intrafractional motion. In the two‐isocenter technique, the isocenter needs to be moved in order to treat the tangents and that may result in intrafraction motion and setup uncertainties in the superior direction.

### E. Comparisons and statistical analyses

Dosimetric comparisons between the two techniques were performed based on the parameters extracted from the dose volume histograms (DVHs): the average volume of the breast CTV receiving 95%, 100%, 105%, and 110% of the prescription dose, maximum dose, uniformity index (UI) (defined as the prescription dose/maximum dose), volume of the ipsilateral lung receiving 20 Gy ($V_{20\text{Gy}}$), volume of the heart receiving 30 Gy ($V_{30\text{Gy}}$), and the volume of the contralateral breast receiving a dose of 1 Gy. All statistical analyses were performed using paired Student's *t*‐test to assess whether the means of two groups were statistically significant. If the probability was 0.05 or less, then the two groups were considered statistically significant.

## III. RESULTS

For all patients, the percentage volume of the breast CTV that received 100% ($V_{100\%}$) of the prescribed dose was 99.3%±0.5% and 96.4%±1.2% for the for two‐isocenter and single‐isocenter plans (p<0.05), respectively. The breast CTV receiving 95% of the prescription dose ($V_{95\%}$) was close to 100% in both techniques. The average percentage volume of the breast CTV receiving 105% of the dose was 32.4%±19.3% for the two‐isocentric technique vs. 23.8%±13.3% for the single‐isocentric technique (p=0.08). The percentage volume of the breast CTV receiving 110% of the dose was 0.4%±1.2% in the two‐isocentric technique vs. 0.1%±0.2% in the single‐isocentric technique. The average uniformity index was 0.91±0.02 vs. 0.91±0.01 in both techniques (p=0.04). Table 2 summaries the dosimetric results for the both techniques. The differences in breast CTV $V_{95\%}$ and $V_{100\%}$ for the breast medium size between the two techniques were statistically significant, but had no clinical impact on the quality of the plans. Breast CTV $V_{100\%}$ and uniformity index were statistically significant, but both plans met treatment planning criteria and were clinically acceptable.

**Table 2:** Uniformity index (UI), $V_{95\%},V_{100\%},V_{105\%}$ and $V_{110\%}$, for breast CTV

		$UI^{a}$				*Breast CTV* $V_{95\%}$				*Breast CTV* $V_{100\%}$				*Breast CTV* $V_{105\%}$				*Breast CTV* $V_{110\%}$			
*Technique/Size*		*S*	*M*	*L*	*All*	*S*	*M*	*L*	*All*	*S*	*M*	*L*	*All*	*S*	*M*	*L*	*All*	*S*	*M*	*L*	*All*
2‐Iso	AVG	0.92	0.91	0.92	0.91	100	100	100	100	99.2	99.5	99.0	99.30	30.8	33.2	32.2	32.4	1.54	0.25	0	0.43
	SD	0.03	0.01	0.01	0.02	0.00	0.00	0.00	0.0	0.3	0.3	0.8	0.52	3.0	12.6	16.8	19.3	2.7	0.4	0.0	1.2
1‐Iso	AVG	0.91	0.91	0.91	0.91	99.9	99.6	99.6	99.69	97.0	95.9	96.7	96.4	20.9	23.6	25.9	23.9	0.18	0.08	0	0.07
	SD	0.01	0.01	0.0	0.01	0.09	0.6	0.5	0.39	1.2	0.6	1.7	1.17	3.4	10.1	18.7	13.3	0.3	0.2	0.0	0.19
p‐value		0.37	0.66	0.05	0.04	0.24	0.08	0.11	0.01	0.12	$2.6\times 10^{-5}$	0.01	$2.6\times 10^{-5}$	0.56	0.17	0.48	0.08	0.42	0.19	n/a	0.22

a ^a^ Uniformity index (UI) is defined as the prescription dose/maximum dose.

S=small; M=medium; L=large separation; AVG=average; $V_{95\%}=\text{volume receiving}\;95\%\;\text{of the prescription dose}$; $V_{100\%}=\text{volume receiving}\;100\%\;\text{of the prescription dose}$; $V_{105\%}=\text{volume receiving}\;105\%\;\text{of the prescription dose}$.

**Figure 3:**
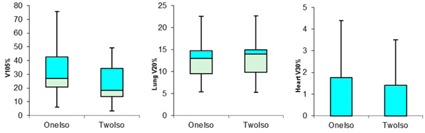
Boxplot comparing the average relative volume of breast CTV receiving 105% of the prescription dose ($V_{105\%}$), the lung average volume receiving 20% of the dose ($V_{20\%}$), and the average volume of heart receiving 30% of the prescription dose ($V_{30\%}$).

**Figure 4:**
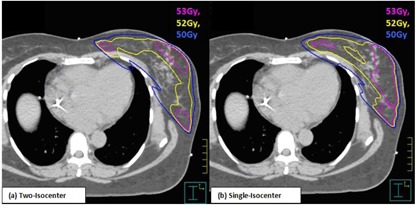
Isodose line distribution for (a) two‐isocenter and (b) single‐isocenter techniques for the same patient.

The percentage of the total lung volume receiving >20 Gy ($V_{20\text{Gy}}$) and the heart receiving >30 Gy ($V_{30\text{Gy}}$) was 13.6% vs. 14.3% (p=0.03) and 1.25% vs. 1.2% (p=0.62), respectively. Table 3 summarizes the $V_{20\text{Gy}}$ for the total lung and the $V_{30\text{Gy}}$ for the heart in both techniques. The difference in lung $V_{20\text{Gy}}$ for medium breast size was statistically significant (p=0.01), but both techniques still satisfy the normal tissue constraint guidelines as recommended by the quantitative analysis of normal tissue effects in the clinic (QUANTEC).^(18)^ The percentage volume of the contralateral breast receiving a dose of 1 Gy was less than 2.3% in small breast patients and insignificant for medium and large breast sizes. Figure 3 shows a “boxplot” analysis for the breast $V_{105\%}$, lung $V_{20\%}$, and heart $V_{30\%}$. The box represents the 25th percentile, 50th percentile, and 75th percentile. Figure 4 shows the isodose lines comparison for the two‐isocentric (left) and one‐isocentric (right) techniques. The delivered dose in both techniques clinically satisfied the breast CTV coverage and normal tissue constraints. The average number of monitor units (MUs) in the single‐isocenter technique was 324.1±99.1 MUs vs. 270.3±92.7 MUs in the two‐isocenter technique (p=0.001).

Shifting the treatment isocenter by 1 mm and 2 mm superiorly showed that the average maximum dose to 1 cc of the breast volume were 55.5±1.8 Gy and 58.6±4.3 Gy in the two‐isocentric technique vs. 56.4±2.1 Gy and 59.1±5.1 Gy in the single‐isocentric technique (p=0.46, 0.87), respectively. Table 4 shows the maximum dose to 1 cc of the normal tissue volume in both techniques. The difference between the two techniques is statistically insignificant, but the single‐isocenter showed higher motion sensitivity.

**Table 3:** Total lung receiving 20Gy ($V_{20\text{Gy}}$), heart volume receiving 30 Gy ($V_{30\text{Gy}}$), and volume of contralateral breast receiving above 1 Gy

		*Lung* $V_{20Gy}\;(\%)$				*Heart* $V_{30Gy}\;(\%)$				Contralateral Breast Volume Receiving>1 Gy (%)			
*Technique/Size*		*S*	*M*	*L*	*All*	*S*	*M*	*L*	*All*	*S*	*M*	*L*	*All*
2‐Iso	AVG	9.3	15.4	11.9	13.6	0.4	1.2	2.8	1.25	1.3	0.7	0.1	0.7
	SD	4.0	6.3	3.2	5.75	0.5	2.5	2.9	2.15	1.2	1.3	0.1	1.15
1‐Iso	AVG	9.9	16.5	11.8	14.3	0.4	1.2	2.7	1.22	2.3	1.0	0.03	1.07
	SD	4.5	6.9	3.8	6.5	0.5	2.4	2.9	2.15	2.2	1.7	0.1	1.71
p‐value		0.28	0.01	0.55	0.03	0.42	0.51	0.59	0.62	0.42	0.08	0.37	0.09

**Table 4:** Maximum dose (average (SD)) to 1 cc of the normal tissue due to the intrafraction motion in the superior direction

*Shifts in the Superior Direction (mm)*	*2‐ISO* $D_{1\text{cc}}$ *(Gy)*	*1‐ISO* $D_{1\text{cc}}$ *(Gy)*	*p‐value*
0	54.23 (0.6)	54.76 (0.2)	0.17
1	55.45 (1.8)	56.41 (2.1)	0.46
2	58.61 (4.3)	59.13 (5.1)	0.87

## IV. DISCUSSION

The purpose of this study was to compare the two‐isocentric technique to the single‐isocentric planning technique for patients receiving breast and supraclavicular lymph nodes irradiation. In the two‐isocenter technique, the patients were treated with either wedged or FIF tangential fields and an oblique anterior SCV field. The single‐isocenter technique was planned using four‐field hybrid IMRT for the tangents and an oblique anterior for the supraclavicular region. Four‐field hybrid IMRT was the choice in this study for the breast tangents, as recommended by Mayo et al.^(14)^ who concluded that the four‐field hybrid is a superior solution.

The single‐isocentric technique eliminates couch and collimator rotations and patient repositioning during treatment, therefore reducing patient setup time and intrafractional patient movement. Adding two more IMRT fields in the single‐isocenter technique increased the number of monitor units by 7%, but the overall treatment time would be reduced significantly by eliminating patient repositioning and couch/collimator rotations. Table 2 shows that both techniques are comparable in breast CTV coverage and uniformity index. Although, the differences in $V_{100\%}$ for medium and large breast CTV are statistically significant between the two but had no clinical significance. Lung $V_{20\text{Gy}}$ for medium breast CTV was statistically significant between the two techniques but had no clinical significance.

The intrafraction motion was simulated only in the superior direction due to the high dose gradient and motion sensitivity in that area. In lateral and vertical directions, the intrafraction motions will have minimum dosimetric impact because of the large margins (flash) in both directions. Table 4 shows that the single‐isocenter with hybrid IMRT is more sensitive to motion than the two‐isocenter technique. This motion sensitivity in hybrid IMRT depends on the number, size, and location of the IMRT segments.

The two‐isocenter method adds other potential sources of errors due to the couch rotation and patient repositioning. Patient repositioning requires the radiation therapists to go inside the room to move the patient, which adds extra time to the treatment. Also, couch and collimator rotation isocentric uncertainties can increase the intrafraction motion of the two‐isocenter technique. These extra sources of errors are avoided in the single‐isocenter method. Therefore, the possibility of intrafraction motion would be higher for the two‐isocentric technique.

## V. CONCLUSIONS

Single isocentric technique using four‐field hybrid IMRT for the tangents and oblique anterior for the supraclavicular field resulted in comparable plan quality as the two‐isocentric technique. Based on our data, if the field size permitted, the single‐isocentric is recommended for treatment of breast cancer patients receiving supraclavicular lymph node irradiation. The single‐isocenter technique increased numbers of MUs slightly, but it eliminated the treatment uncertainties due to the couch and collimator rotations, and also reduced patient setup time.

Our study results were applied to breast patients treated without respiratory gating. If gating is used to reduce the dose to the heart, then using the single‐isocenter technique may benefit the patient by reducing the setup uncertainties and repositioning the isocenter during treatment. Sensitivity to intrafraction motion will be higher in case of gating and should be studied in all directions.
